# Cell therapies for chondral defects of the talus: a systematic review

**DOI:** 10.1186/s13018-022-03203-4

**Published:** 2022-06-11

**Authors:** Filippo Migliorini, Jörg Eschweiler, Christian Goetze, Torsten Pastor, Riccardo Giorgino, Frank Hildebrand, Nicola Maffulli

**Affiliations:** 1 Department of Orthopaedic, Trauma, and Reconstructive Surgery, RWTH University Hospital, Pauwelsstraße 30, 52074 Aachen, Germany; 2 Department of Orthopaedic Surgery, Auguste-Viktoria Clinic, Ruhr University Bochum, 32545 Bad Oeynhausen, Germany; 3 Department of Orthopaedic and Trauma Surgery, Cantonal Hospital, 6000 Lucerne, Switzerland; 4 IRCCS Istituto Ortopedico Galeazzi, University of Milan, 20161 Milan, Italy; 5 Department of Medicine, Surgery and Dentistry, University of Salerno, 84081 Baronissi, Italy; 6 Faculty of Medicine, School of Pharmacy and Bioengineering, Keele University, ST4 7QB Stoke on Trent, England; 7 Barts and the London School of Medicine and Dentistry, Centre for Sports and Exercise Medicine, Mile End Hospital, Queen Mary University of London, E1 4DG London, England

**Keywords:** Ankle, Cartilage defects, Mesenchymal stem cells, Autologous chondrocyte implantation

## Abstract

**Background:**

This systematic review investigated the efficacy and safety of surgical procedures augmented with cell therapies for chondral defects of the talus.

**Methods:**

The present systematic review was conducted according to the 2020 PRISMA guidelines. PubMed, Google scholar, Embase, and Scopus databases were accessed in March 2022. All the clinical trials investigating surgical procedures for talar chondral defects augmented with cell therapies were accessed. The outcomes of interest were to investigate whether surgical procedures augmented with cell therapies promoted improvement in patients reported outcomes measures (PROMs) with a tolerable rate of complications.

**Results:**

Data from 477 procedures were retrieved. At a mean follow-up of 34.8 ± 9.7 months, the Visual Analogic Scale (VAS) improved of 4.4/10 (*P* = 0.002) and the American Orthopaedic Foot and Ankle Score (AOFAS) of 31.1/100 (*P* = 0.0001) points. No improvement was found in Tegner score (*P* = 0.4). Few articles reported data on complications. At last follow-up, the rate of reoperation and failure were 0.06% and 0.03%, respectively. No graft delamination or hypertrophy was observed.

**Conclusion:**

The current evidence suggests that cell therapies may be effective and safe to enhance surgical procedures for chondral defects of the talus. These results should be considered within the limitations of the present study. The current literature should be enriched with randomized controlled clinical trials with larger population size and longer follow-up.

## Introduction

Focal chondral defects of the talus are common in the young and active population [1, 2]. Given the limited healing potential of hyaline cartilage, these lesions are most likely unable to regenerate [3, 4]. If left untreated, patients may experience chronic instability, persistent pain, and early onset osteoarthritis [5–7]. Defects smaller than 0.5 cm^2^ can be managed arthroscopically with microfractures (MFx), and several procedures have been advocated for bigger defects [8–12]. Autologous chondrocyte implantation (ACI), osteochondral transplantation, Autologous Matrix-Induced Chondrogenesis (AMIC) have been advocated for chondral defects of the talus [13–17]. Although results are promising, the rate of failure of these procedures is between 1 and 10% [18–23]. Chondral procedures can be enhanced with cell therapies to augment the healing process, increase the regeneration potential, and reduce fibrosis [24–28]. Cell therapies to enhance chondral repair of the talus evolved in the past two decades. Several preclinical studies have been published, while clinical investigations are still limited. Surgical augmentation with mesenchymal stem cells (MSCs) for chondral repair developed recently [18, 29, 30]. These procedures are mainly based on MSCs or bone marrow aspirate concentrate (BMAC) transplantation [31–33]. Chondral procedures combined with stem cells therapies are obtained, processed, and delivered during a relatively simple one-step procedure [25–27, 34, 35]. The delivery cells rich in growth factors and chemokines to enhance cell migration and proliferation represent another commonly used type of cell therapy [36–38]. In this regard, the injection of platelet rich plasma (PRP) and/or its derivate growth factors, or simple peripheral blood injections also gained interest in the past decades [39–43]. The current literature reports several clinical investigations which evaluated the efficacy and safety of cell therapies augmentation for chondral defects of the talus. However, to the best of our knowledge, a comprehensive systematic review is lacking. We therefore updated the current available evidence and investigated the efficacy and safety of cell therapies augmentation for chondral defects of the talus.

## Materials and methods

### Search strategy

This systematic review was conducted according to the Preferred Reporting Items for Systematic Reviews and Meta-Analyses (PRISMA) checlist [44] and the guidelines form the Cochrane Handbook for Systematic Reviews of Interventions [45]. Two authors (F.M. and J.E.) independently performed the literature search in March 2022 accessing PubMed, Google scholar, Embase, and Scopus databases. The following keywords were used in combination using the Boolean operators AND/OR: *(talus)* AND *(chondral defects* AND *focal)* AND *(management* OR *surgery* OR *therapy* OR *arthroscopy)* AND *(pain* OR *sports* OR *ACI* OR *autologous chondrocyte implantation* OR *matrix-induced* OR *periosteum* OR *membrane* OR *chondral* OR *collagen* OR *visual analogic scale* OR *PROMs* OR *patient reported outcome measures* AND *((blood* OR *mesenchymal* AND *stem (cells* OR *cell)* AND *(concentrate* OR *application* OR *augmentation* OR *enhancement)* AND *bone marrow* OR *adipose* OR *peripheral blood)).* No further filters, keywords, or limits were used for the databases search. No time constrains were used for the search. The same framework was used in each database. The two reviewers independently screened the resulting titles per hand. If titles matched the topic, the abstract was accessed. Titles which did not focus the main topic were excluded. The two reviewers independently screened the resulting abstract by hand. For those abstract which could potentially match the topic, the full text article was downloaded. The two reviewers independently evalauted the bibliographies of the full text articles. In a second phase, all the articles were listed aphabetically according to the surname of the first author and year of pubblication in Microsoft Excel (version MacOS 16.37, Microsoft Corporation, USA). Duplicates were excluded. Discrepancies were further evaluated, and debated by both reviewers and disagreements were solved by a third author (N.M.).

### Inclusion criteria

All the published literature investigating procedures to address chondral defects of the talus augmented with cell therapies were accessed. Given to the authors’ language capabilities, articles in English, German, Italian, French and Spanish were eligible. Studies with level I to IV of evidence, according to Oxford Centre of Evidence-Based Medicine [46], were considered. Only clinical investigations which focused on chondral defects of the talus were eligible. Only clinical studies published in peer reviewed journals were considered. Studies reporting data on cell therapies augmentation for ACI, osteochondral allograft and autograft transplantation, and AMIC were eligible. Only articles reporting quantitative data under the outcomes of interest were considered for inclusion.

### Exclusion criteria

Studies reporting data on patients who underwent chondral procedures in participants with advanced degenerative chondropathy were not eligible. In vitro, animal, and computational studies were not considered. Studies which augmented chondral procedures with less differentiated stem cells (e.g. totipotential, multipotential, humbelical) or fully committed cells (e.g. chondrocytes) were not considered. Studies reporting data on patient who underwent allogenic or xenogenic cells were not considered. Missing data under the outcomes of interest warranted the exclusion from this study.

### Data extraction and outcomes of interest

Two authors (F.M. and J.E.) independently performed data extraction. Study generalities (author, year, journal, type of study, length of the follow-up) and patients baseline characteristics (number of procedures, mean BMI, mean age, mean length of the symptoms before surgery, percentage of women, and mean size of the defect) were collected. Data from the following patient reported outcome measures (PROMs) were collected at baseline and at last follow-up: Visual Analogic Scale (VAS), Tegner Activity Scale [47], and American Orthopaedic Foot and Ankle Score (AOFAS) [48]. The rate of graft hypertrophy or delamination was retrieved, as was the rate of failure and revision surgery. We investigated whether PROMs improved from baseline to the last follow-up, and reported the frequency of complications.

### Methodology quality assessment

The methodological quality assessment was performed by one author (F.M.) following the guidelines of the Cochrane Handbook for Systematic Reviews of Interventions [45]. The risk of bias graph tool of the Review Manager Software Version 5.3 (The Nordic Cochrane Collaboration, Copenhagen) was used. The following bias were considered: selection, detection, attrition, reporting, and other source of bias. The risk of selection bias analysed the random sequence generation and the allocation concealment. The risk of detection bias in the blinding procedure during the outcome assessment were analysed. The risk of attrition bias refers to incomplete outcome data, such as missing outcome data from attrition during study enrollment or analysis. The risk of reporting bias refers to the selective publication of results based on the their statistical or clinical relevance. If the authors indentified additional risk of bias, these were considered as “other bias”. The risk of bias tool evalautes each bias as low (green), high (red), or unclear (yellow). The risk associated to each bias is expressed as percentage. The quality of evidence of collective outcomes were evaluated using the Grading of Recommendations, Assessment, Development, and Evaluation (GRADE) system was used [49, 50].

### Statistical analysis

The statistical analyses were performed by the main author (F.M.) using the IBM SPSS software (version 25). For descriptive statistics of continuous endpoint, mean and standard deviation was evaluated. For binary data (rate of failure, revision surgery, graft hypertrophy and delamination), the number of observations and the number of patients for each study were collected. To evaluate the improvement of PROMs from baseline to the last follow-up, the mean difference (MD) was calculated, with P values of t-test < 0.05 considered statistically significant.

## Results

### Search result

**Fig. 1:**
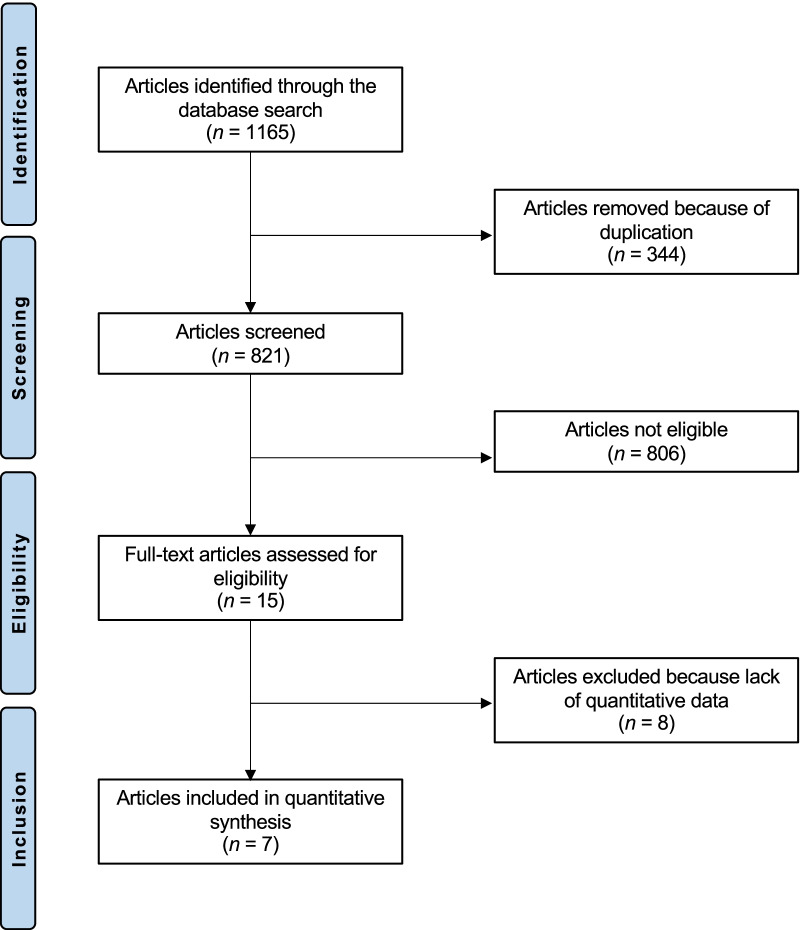
Flow-chart of the literature search

The literature search resulted in 1165 articles. Of these, 344 were duplicates. A further 806 studies were excluded: not clinical studies (N = 307), not focusing on talus (N = 279), study design (N = 101), evaluating other procedures rather than ACI, osteochondral allograft and autograft transplantation, or AMIC (N = 74), reporting data on patients with advanced degenerative chondropathy (N = 2), not published in peer reviewed journal (N = 19), using less differentiated stem cells, fully committed cells, allogenic or xenogenic cells (N = 21), language limitation (N = 3). A further eight studies were not included as they did not report quantitative data under the outcomes of interest. Finally, 7 articles were included in the present study. The results of the literature search are shown in Fig. 1.

### Methodological quality assessment

**Fig. 2:**
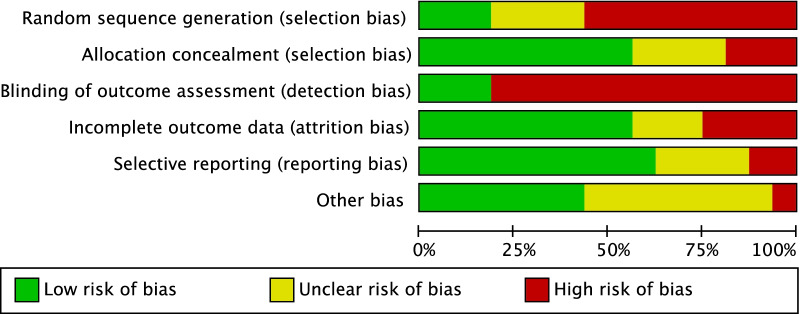
Methodological quality assessment. The risk of bias tool assessed the risk of bias (low, unclear, or high) per each risk of bias item presented as percentages across all included studies

**Fig. 3:**
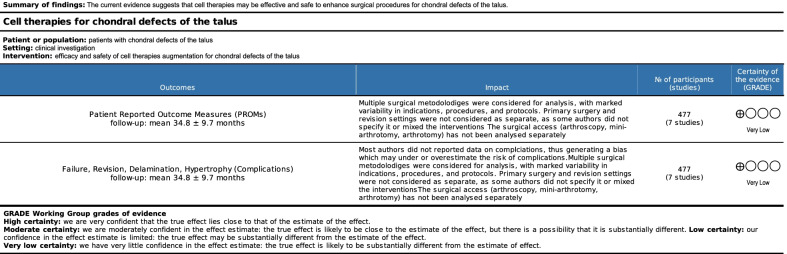
GRADE

The retrospective design in 43% (3 of 7) of the included studies increases the risk of selection bias which was scored as moderate. Moreover, most of the included studies did not adopt a blinding method, thus increasing the risk of detection bias. Overall, the missing outcome data from attrition during study enrollment or analysis was low, leading to a low to moderate attrition bias. The risk of reporting bias was moderate, and the risk of other biases moderate to high. Concluding, the overall quality of the methodological assessment was moderate (Figs. 2, 3).

### Patient demographics

Data from 477 procedures were retrieved. The mean duration of symptoms before the index surgery was 6.6 ± 4.6 months. The mean follow-up was 34.8 ± 9.7 months. The mean age of the patients was 34.2 ± 6.7 years. 32% (153 of 477 patients) were women. The mean defect size was 1.7 ± 0.4 cm^2^. Few studies reported data with regard to the location of the lesion: medial 73% (99 of 135), lateral 27% (36 of 135), central 0% (0 of 135). Patient demographics at baseline is shown in greater detail in Table 1 (BMC—bone marrow concentrate; PBC—peripheral blood concentrate; MAST—matrix-associated stem cell transplantation; AMIC—autologous matrix-induced chondrogenesis; PRP—platelet-rich plasma; mACI—membrane-assisted autologous chondrocyte implantation; PRGF—platelet-rich grow factors) (Table 2).

**Table 1:** Generalities and patient baseline of the included studies

Author, year	Journal	Study Design	Follow-up (months)	Treatment	Procedures (n)	Female (%)	Mean age	Mean BMI	Mean defect size (cm^2^)
Buda et al. [51]	*Int Orthop*	Retrospective	48.0	Control group (mACI)	40	37.5	31.4		1.7
				BMAC	40	32.5	30.2		1.8
Desando et al. [52]	*Cartilage*	Prospective	36.0	Control group (mACI)	7	42.9	31.2		1.8
			36.0	BMAC	15	33.3	31.0		
Guney et al. [53]	*Knee Surg Sports Traumatol Arthrosc*	Prospective	47.3	Control group (MFX)	19	37.4	47, 4		
			40.4	MFX & PRP	22	43.9	50.0		
			30.1	Control group (Mosaicplasty)	13	37.6	15.4		> 0.2
Murphy et al. [54]	*Knee Surg Sports Traumatol Arthrosc*	Retrospective	36.7	MAST	38	31.2	35.0		1.7
Nguyen et al. [55]	*Am J Sports Med*	Retrospective	44.7	OAT & BMA, or PRGF	38	0.0	26.0		2.5
Richter et al. [56]	*Foot Ankle Surg*	Prospective	24.0	MAST & BMC	26	28.0	33.0		1.1
Richter et al. [57]	*Foot Ankle Surg*	Prospective	24.4	MAST & BMC	129	41.0	35.3		1.6
			23.8	AMIC & PBC	129	40.0	35.6		1.8

*BMC*—bone marrow concentrate, *PBC*—peripheral blood concentrate, *MAST*—matrix-associated stem cell transplantation, *AMIC*—autologous matrix-induced chondrogenesis, *PRP*—platelet-rich plasma, *mACI*—membrane-assisted autologous chondrocyte implantation, *PRGF*—platelet-rich grow factors

**Table 2:** Results of patient reported outcome measures (PROMs)

Endpoint	Baseline	Last follow-up	MD	*P*
VAS	6.8 ± 2.1	2.4 ± 1.7	− 4.4	0.002
Tegner	5.0 ± 2.8	5.5 ± 0.7	0.5	0.4
AOFAS	54.1 ± 6.7	85.2 ± 7.2	31.1	0.0001

*VAS*—visual analogue scale, *AOFAS*—American Orthopaedic Foot and Ankle Score

*P* values of t-test < 0.05 are considered statistically significant

### Outcomes of interest

At last follow-up, the VAS had improved of -4.4/10 (*P* = 0.002) and the AOFAS of 31.1/100 (*P* = 0.0001). No improvement was found in Tegner score (*P* = 0.4).

### Complications

Few articles reported data on complications. At last follow-up the rates of reoperation and failure were 0.06% and 0.03%, respectively. No graft delamination or hypertrophy were observed. Table 3 shows the frequency of complication (data are based on the total number of patients that were included in the articles reporting quantitative data on such complication).

**Table 3:** Frequency of complication (data are based on the total number of patients that were included in the articles reporting quantitative data on such complication)

Endpoint	Rate
Reoperation	5.2% (19 of 362)
Delamination	0% (0 of 284)
Hypertrophy	0% (0 of 284)
Failures	3.3% (12 of 362)

### Quality of the recommendations

The overall quality of evidence of collective outcomes according to the GRADE approach was very low (Fig. 4).

## Discussion

According to the main findings of the present study, cell therapies augmentation for surgical procedures may enhance cartilage regeneration in chondral defects of the talus. PROMs were significant improved from baseline to the last follow-up, indicating that these procedures may be effective in restoring ankle function, reducing the symptoms and improving the physical activity of the patients. The risk of reoperations and failures were 5.2% and 3.3%, respectively, and no delamination or hypertrophy were evidenced at last follow-up, indicating that these procedures may be safely performed.

The type of surgical procedures and cell therapies used for augmentation were heterogeneous. Guney et al. [53] enhanced MFx with platelet rich plasma (PRP) 6 to 24 h after the arthroscopic procedure. They found an improvement of the AOFAS and VAS scores at a mean of 42 months follow-up [53]. However, they reported greater pain control in the control group (mosaicplasty) [53]. PRP is obtained by centrifugation of platelets extracted by peripheral venous blood [58, 59]. PRP was introduced in the early 50’s: since then, it has been employed in regenerative medicine, and extended to musculoskeletal disorders [58, 60, 61]. PRP has a high concentration of growth factors, such as TGF-β, VEGF, EGF, IGF-1, b-FGF, [62, 63]. These growth factors and mediators enhance chemotaxis, angiogenesis, cell proliferation, and matrix formation of MSCs, accelerating tissue heling and improve regeneration [63–68]. Moreover, previous studies found that PRP reduces catabolism and increases the anabolic activity of hyaline cartilage [69]. Given its regenerative potential, PRP has been advocated in the conservative management of several musculoskeletal ailments [69–72]. Nguyen et al. [55] augmented osteochondral autograft transplantation (OAT) with bone marrow aspirate concentrate or platelet-rich grow factors in a cohort of 38 athletes. 87% (33 of 38) of athletes returned to sport at their previous level within a mean of 8.2 months, 11% (4 of 38) returned at a lower level, and 2% (1 of 38) did not return to sport [55]. Although they did not report data separately according to their augmentation procedure, overall good outcome and patient satisfaction were observed [55].

Most of the cell therapy modalities included in the present study promoted MSCs migration and proliferation [51, 52, 54–57]. In addition to their cellular differentiation potential, the paracrine activity of MSCs interact with the microenvironment, enhancing tissue regeneration and modulating inflammation, promoting T and B cell proliferation, and NK cell activity [25, 26, 73]. The power of regenerative medicine relies in the signalling and mutual interaction patterns between stem cells and environment [74–76]. MSCs can act as pericytes, releasing factors with reparative, anti-inflammatory, and immunomodulatory effect [25, 77]. These characteristics suggest that, beyond their interation with the environment and differentiation potential, MSCs also modulate inflammation, which is pivotal in pain control. MSCs release trophic, anabolic, and chemotactic cytokines which attract further MSCs to the defect, enhancing neocartilage integration and collagen type II expression ^78^. This function of MSCs as chemofactors and supervisors is essential for tissue repair and regeneration. Richter et al. [57] compared 129 patients who underwent matrix-associated stem cell transplantation (MAST) *versus* 129 patients who underwent AMIC augmented with peripheral blood concentrate. At two-year follow-up, they evidenced no difference between the two procedures in the rate of revision and failures [57]. Isolated AMIC for chondral defects of the talus reported promising outcomes [79, 80], with results superior to isolated MFx [81].

This study certainly has limitations. The limited number of included studies and the retrospective design of most of studies represent an important weakness of the present investigation. However, we point out that such limitations are intrinsic of the published scientific literature, which lacks high level of evidence investigations. Inter-rater agreement during study selection has not been conducted. The evidence on surgical strategies for chondral defects of the talus augmented with cell therapies is limited. Multiple surgical metodolodiges were considered for analysis, with marked variability in indications, procedures, and protocols. This surely leads to greater risk of bias, and hence the results from the present study should be considered cautiously. Given the heterogeneous nature of the treatments, along with the limited quantitative data available for inclusion, no further subgroup analyses were conducted. The nature of the membrane used to coat the cells was also heterogeneous: some author used a hyaluronic acid based membrane [51, 52], other a collagen I/III porcine derived scaffold [54, 56, 57]. Primary surgery and revision settings were not considered as separate, as some authors did not specify it or mixed the interventions. Only Guney et al. [53] considered solely primary interventions. The authors of the included studies did not adequately specify whether the lesions were acute or chronic, or considered them separately in the analyses. Chondral damage of the talus is typically caused by an acute injury, such as a sudden pivot or twist, a fall, or direct blow to the ankle [82]. Less common causes have been described, such as prolonged immobilization, osteochondritis, alteration in the forces exerted on the articular cartilage, nutritional inadequacies [83, 84]. The surgical access (arthroscopy, mini-arthrotomy, arthrotomy) has not been analysed separately. A recent systematic review compared 421 arthroscopic versus 349 mini-arthrotomy approach for mACI in the knee, with no difference between the two groups in Tegner, Lysholm, and International Knee Documentation Committee (IKDC) Score, and in the rate of failures and revisions [85]. PRP preparation and processing protocols have not been yet established. The initial whole blood volume, centrifugation rate, and duration of centrifugation is heterogeneous, and no consensus has been reached [86–91]. Evidence in support of the use of an activator for PRP (calcium chloride) is limited, and its implementation unclear [92, 93]. The best method to enhance surgical procedures addressing chondral defect is still unknown. Standardization of surgical procedures, methods of cell harvesting and delivery, and timing of outcome measures assessment must be better standardised. To optimize the performance of chondral procedures augmented with cell-based therapies, stricter eligibility criteria for such techniques must be also clarified. The current literature should be enriched with randomized controlled clinical trials with larger population size and longer follow-up times.

## Conclusions

The current evidence suggests that cell therapies may be effective and safe to enhance surgical procedures for chondral defects of the talus. These results should be considered within the limitations of the present study. The current literature should be enriched with randomized controlled clinical trials with larger population size and longer follow-up.

## Data Availability

The datasets generated during and/or analysed during the current study are available throughout the manuscript.

## Abbreviations

MFx: Microfractures
ACI: Autologous chondrocyte implantation
OAT: Osteochondral allograft and autograft transplantation
BMAC: Stem cells (MSC) or bone marrow aspirate concentrate
IKDC: International Knee Documentation Committee
MD: Mean difference
BMC: Bone marrow concentrate
PBC: Peripheral blood concentrate
PRP: Platelet-rich plasma
mACI: Membrane-assisted autologous chondrocyte implantation
PRGF: Platelet-rich grow factor
MAST: Matrix-associated stem cell transplantation
AMIC: Autologous matrix-induced chondrogenesis
